# The impact of a complete nutrition diabetic formula on glycemic control and nutritional status in hospitalized patients with diabetes mellitus

**DOI:** 10.3389/fendo.2025.1629741

**Published:** 2025-07-01

**Authors:** Chin-Kun Wang, Ke-Xin Li, Sheng-Qian Sun, Tristan-C. Liu

**Affiliations:** ^1^ Department of Nutrition, Chung Shan Medical University, Taichung, China; ^2^ Le Bonta Wellness Co. Ltd, Shanghai, China; ^3^ Research and Development Center, Standard Investment (China) Ltd., Shanghai, China

**Keywords:** diabetes, oral nutritional supplements (ONS), glycemic control, nutritional status, mid-arm circumference (MAC)

## Abstract

The clinical application of specialized oral nutritional supplements (ONS) in managing hospitalized patients with diabetes mellitus necessitates rigorous evaluation of their effects on glycemic stability and nutritional parameters. This prospective study aimed to investigate the impact of a diabetes-specific ONS, Complete Nutrition Diabetic Formula, on glycemic control and key nutritional indices in diabetic inpatients. Forty adult inpatients diagnosed with diabetes mellitus requiring nutritional support were enrolled and completed the intervention. Following assessment of individual caloric requirements, participants received an average daily supplementation of 156 g (equivalent to 2.6 packs) of the Complete Nutrition Diabetic Formula over a mean duration of 29 days (range 14-67 days). Glycemic parameters, anthropometric measurements (including mid-arm circumference, MAC), and pertinent biochemical markers (including serum magnesium) were assessed at baseline and study conclusion. Post-intervention analysis showed a higher total caloric and fat intake with a lower carbohydrate share, accompanied by significant gains in mid- arm circumference (MAC), serum magnesium, and a nitrogen-balance flip from -2.7 g/day to +1.25 g/day (*p* < 0.05), marking a catabolic - to - anabolic shift and confirming protein repletion. Fasting plasma glucose and other biochemical indices - including serum proteins and lipid profiles - remained unchanged (*p* > 0.05), indicating that short-term Complete Nutrition Diabetic Formula supplementation improves key nutritional indicators without compromising glycemic control or broader metabolic stability.

## 1 Introduction

Diabetes mellitus represents a significant and escalating global health burden, imposing substantial demands on healthcare systems worldwide. Within the hospital environment, patients with diabetes face a compounded challenge due to a remarkably high prevalence of malnutrition. Recent meta-analyses indicate that approximately one-third (33%) of individuals with diabetes suffer from malnutrition, with an additional 44% being identified as at risk (1). This risk is particularly pronounced among hospitalized elderly patients with diabetes, where studies report malnutrition or risk thereof affecting up to 60% of this cohort (2). The issue extends beyond the inpatient setting, as nutritional risk is frequently identified even among diabetic patients attending outpatient clinics (3).

This high prevalence of malnutrition among hospitalized diabetic patients is intrinsically linked to a cascade of adverse clinical outcomes. Malnutrition in this population contributes significantly to increased morbidity, impairs essential physiological processes such as muscle function and wound healing, compromises immune function, and leads to general functional decline. Furthermore, it is associated with prolonged hospital stays, increased rates of hospital readmission, elevated healthcare expenditures, and ultimately, significantly worsened survival rates (2). The presence of diabetes itself is an independent risk factor for poorer clinical outcomes across various medical conditions; the co-existence of malnutrition markedly exacerbates this risk, creating a highly vulnerable patient subgroup (2).Nutritional assessment in diabetic patients is complicated by the frequent co-occurrence of overweight or obesity. This common clinical presentation can paradoxically mask underlying malnutrition, particularly deficits in lean body mass (sarcopenia) or specific micronutrient deficiencies, which are not always reflected by traditional metrics like Body Mass Index (BMI). Indeed, studies have shown that malnutrition risk in elderly diabetic patients is often independent of their BMI. Consequently, relying solely on weight or BMI for nutritional screening is insufficient in this population (4), necessitating more comprehensive assessment strategies. This underscores the importance of evaluating parameters like mid-arm circumference (MAC), which may better reflect muscle status irrespective of overall body weight or fluid balance (5).

Moreover, the concept of malnutrition encompasses a spectrum of nutritional derangements, including deficiencies in specific micronutrients, altered metabolism secondary to acute or chronic disease, and impaired nutrient absorption or utilization. Diabetes itself is associated with disturbances in micronutrient homeostasis, such as altered magnesium metabolism (6). Therefore, addressing malnutrition effectively requires interventions that provide not only adequate macronutrients but also sufficient micronutrients. A major clinical challenge lies in providing this essential nutritional support while simultaneously maintaining adequate glycemic control, as hyperglycemia itself is a frequent complication during nutritional therapy (7) and is independently associated with adverse outcomes, including increased risk of infections and mortality (8). Medical nutrition therapy is a cornerstone in the management of hospitalized patients, particularly for those with diabetes identified as malnourished or at nutritional risk. Timely and adequate nutritional support, delivered via enteral or parenteral routes, is crucial for mitigating the detrimental consequences of malnutrition and supporting recovery (8).When the gastrointestinal tract is functional, enteral nutrition (EN) is the preferred route over parenteral nutrition (PN) (9). This preference is based on EN’s physiological benefits, including maintaining gut integrity, potentially reducing infectious complications, and, critically for diabetic patients, often resulting in less pronounced hyperglycemia compared to PN (10). The more significant glycemic excursions observed with PN are partly attributable to bypassing the gastrointestinal tract and the associated incretin effect (11).

Effective management necessitates achieving appropriate glycemic targets. Consensus guidelines generally recommend maintaining blood glucose levels within a target range of 140–180 mg/dL (7.8–10.0 mmol/L) for the majority of hospitalized patients receiving nutritional support, balancing glycemic control with hypoglycemia risk (10). To address the challenge of managing blood glucose during EN in diabetic patients, diabetes-specific formulas (DSFs) have been developed. These formulations differ from standard enteral formulas (SFs), typically featuring a modified carbohydrate profile (lower total content, often 35-50% vs. 50-60% in SFs; low-glycemic index (GI) sources like fructose, isomaltulose, modified starches) and increased dietary fiber. DSFs also frequently contain a higher proportion of fat, particularly monounsaturated fatty acids (MUFAs) (12). This composition aims to attenuate postprandial glucose rise.

Systematic reviews and meta-analyses consistently demonstrate DSFs’ efficacy in improving glycemic control compared to SFs. These studies report significant reductions in postprandial glucose excursions, peak blood glucose, and glucose area under the curve (AUC) (12). Some studies also indicate improvements in longer-term markers like HbA1c and fasting blood glucose (13), and potentially reduced insulin requirements. While glycemic benefits are well-established, effects on lipid profiles appear less consistent, with some meta-analyses reporting no significant differences in total cholesterol, LDL, or triglycerides, but potential increases in HDL (12).

Evaluating the impact on overall nutritional status is crucial, given the high malnutrition risk. Mid-Arm Circumference (MAC) is a simple, non-invasive bedside measure indicating peripheral tissue reserves. Its utility is enhanced in hospitalized patients where fluid shifts can confound weight/BMI. MAC is less susceptible to these shifts, offering a potentially more reliable assessment of somatic stores. Studies validate MAC against tools like Subjective Global Assessment (SGA) and show its predictive value for outcomes like length of stay and mortality (5). Magnesium deficiency is also prevalent in type 2 diabetes, particularly with poor glycemic control. Low intracellular magnesium impairs insulin action and worsens insulin resistance. Factors include increased urinary losses and low dietary intake. Hospitalization can exacerbate this due to illness, stress, and medications. While serum magnesium may not fully reflect total body stores, monitoring its changes remains clinically accessible to assess interventions (14).

While the general efficacy of DSFs for glycemic control is established, and individual nutrients like magnesium show potential benefits, a gap remains regarding the integrated effects of specific, complete formulas. There is limited data evaluating the simultaneous impact of a particular complete DSF on both glycemic control and key nutritional status indicators (MAC, serum magnesium) in hospitalized diabetic patients. Large trials on general nutritional support have shown benefits, but subgroup analyses in diabetes yielded inconclusive mortality results, despite positive trends (8), suggesting a need for more targeted approaches or larger trials in this specific group. Furthermore, despite glycemic guidelines (10), routine DSF use recommendations are less definitive and not universally implemented, possibly due to lack of conclusive evidence on overall efficacy and safety beyond glycemia. Investigating the combined effects of a specific formula is essential for evidence-based management.

Given the high prevalence of malnutrition, challenges in glycemic management, and evidence gaps regarding specific DSFs in hospitalized diabetic patients, this study aimed to investigate the effects of supplementation with ‘Complete Nutrition Diabetic Formula’ on nutritional status (MAC, serum magnesium, nitrogen balance) and glycemic control (fasting blood glucose) in this population.

## 2 Materials and methods

### 2.1 Study population and ethical considerations

**Figure 1:**
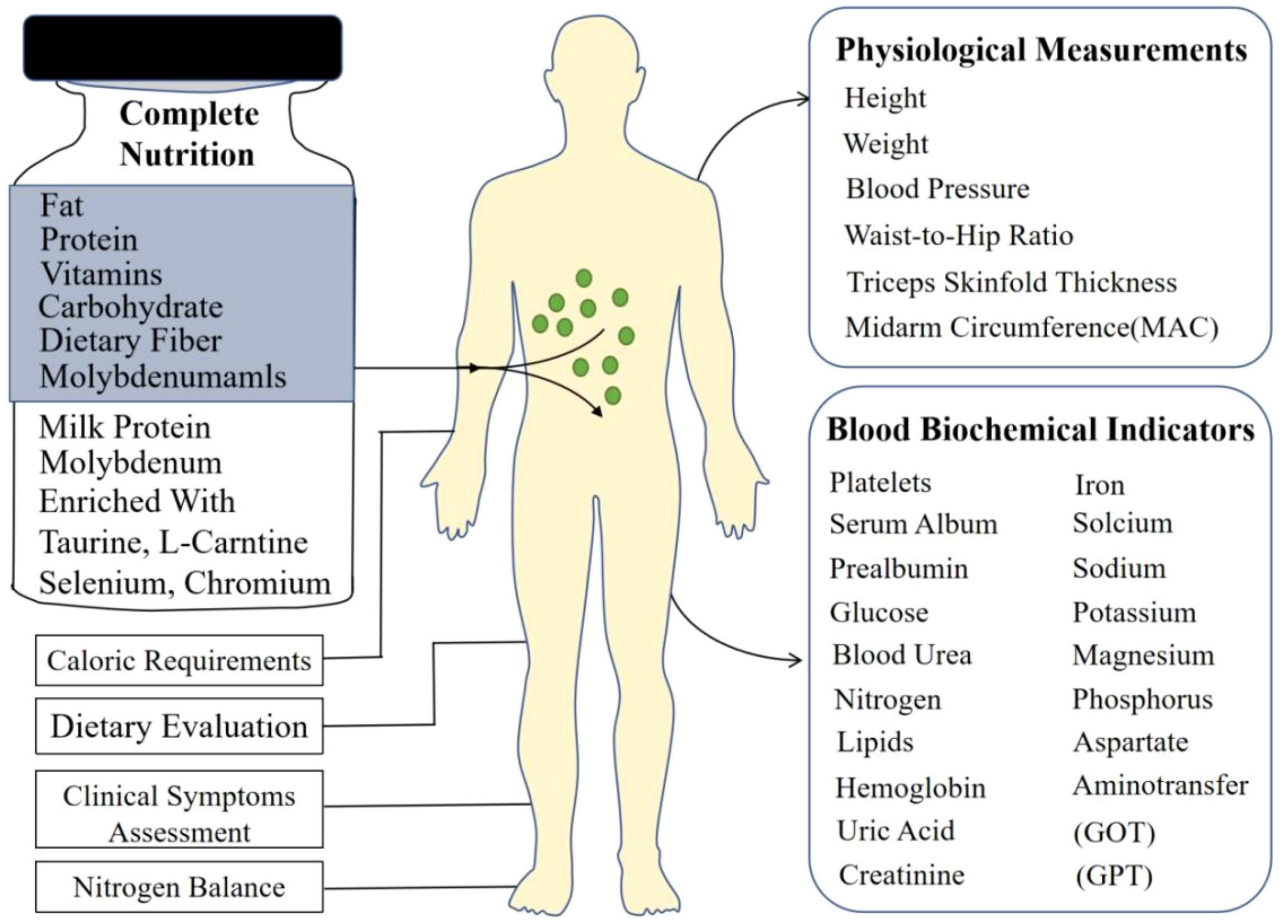
Schematic diagram of the clinical intervention and assessments.

This prospective interventional study was conducted at Chung Shan Medical University Hospital, Taiwan. A total of 40 adult inpatients diagnosed with diabetes mellitus were enrolled and completed the study (19 males, 21 females; mean age 63 years, range 32-81 years). Participants were included if they required nutritional support and consented to the use of a nutritional supplement, irrespective of their baseline nutritional status as assessed by standard clinical criteria. The overall study process, including the nutritional intervention and key assessments performed, is schematically illustrated in **Figure 1**. The inclusion criteria encompassed patients requiring dietary management for glycemic control, including individuals diagnosed with type 1 diabetes, type 2 diabetes, stress-induced hyperglycemia, or other forms of abnormal glucose metabolism necessitating nutritional intervention during hospitalization. Prior to enrollment, all participants provided written informed consent after receiving a detailed explanation of the study’s purpose, procedures, potential risks, and benefits. The study protocol adhered to the principles outlined in the Declaration of Helsinki and received formal approval from the Institutional Review Board (IRB: CS05073) of Chung Shan Medical University Hospital.

### 2.2 Complete nutrition diabetic formula

The nutritional supplement utilized in this investigation was the ‘Complete Nutrition Diabetic Formula’, commercially produced by Standard Foods Corporation (Taiwan). This formula is specifically designed for individuals with diabetes. Each serving consists of 60 grams of powder, which, when reconstituted with water to a final volume of 200 mL, provides 238 kilocalories (kcal). The macronutrient composition per serving is characterized by 20.2% of energy derived from protein, 30.2% from fat, and 49.6% from carbohydrates. This distribution aligns with general nutritional recommendations for diabetic patients. According to the manufacturer, consumption providing 1500 kcal daily meets the recommended dietary allowances for essential macro- and micronutrients (including vitamins and minerals) for Taiwanese adults. Key compositional features relevant to diabetes management include the absence of sucrose (sweetened primarily with sucralose), the inclusion of high-quality milk proteins, 5.9 grams of dietary fiber per serving, and ~17% of fat content as medium-chain triglycerides (MCTs). The formula also contains taurine, L-carnitine, and essential trace elements such as selenium, chromium, and molybdenum. The product is suitable for individuals following a lacto-vegetarian diet.

### 2.3 Experimental procedures

Upon enrollment and baseline assessment, each participant underwent a comprehensive evaluation. Systematic anthropometric assessments were performed at baseline and endpoint by trained personnel. Height was measured using a stadiometer, and body weight was obtained using a calibrated scale; for patients presenting with edema or ascites, estimated dry weight was calculated and utilized for subsequent analyses. Mid-arm circumference (MAC) was measured in centimeters using a non-stretchable tape at the midpoint between the acromion process and the olecranon process of the non-dominant arm, with the mean of three consecutive measurements recorded for accuracy (10). Triceps skinfold thickness (TSF) was measured using calibrated skinfold calipers. Mid-arm muscle circumference (MAMC), an indicator of upper arm muscle mass, was calculated from MAC and TSF values using standard equations. Waist and hip circumferences were measured with a non-stretchable tape at standardized anatomical landmarks. From these primary measurements, Body Mass Index (BMI) was calculated as weight in kilograms divided by height in meters squared (kg/m^2^), and the waist-to-hip ratio (WHR) was derived.

#### 2.3.1 Caloric requirement estimation

Individualized daily energy requirements for each participant were estimated using the Harris-Benedict equation. This calculation incorporated the individual’s basal metabolic rate (BMR), which was further adjusted based on estimated activity level and relevant clinical stress factors associated with their inpatient status to determine total daily energy needs.

#### 2.3.2 Biochemical analyses

Fasting venous blood samples were collected at baseline (pre-supplementation) and at the study endpoint (post-supplementation) for comprehensive biochemical evaluation. Laboratory analyses encompassed hematological parameters via a complete blood count (including red blood cells, white blood cells, platelets, and hemoglobin concentration). Serum proteins, specifically albumin and prealbumin, were measured as indicators of visceral protein status. Glycemic control was assessed via fasting plasma glucose. Renal function was evaluated using blood urea nitrogen (BUN) and serum creatinine levels. A full lipid profile, including total cholesterol, triglycerides, HDL-cholesterol, and LDL-cholesterol, was determined. Electrolyte and mineral concentrations measured included serum sodium, potassium, calcium, phosphorus, serum iron, uric acid, and, crucially for this investigation, serum magnesium. Liver function was assessed through aspartate aminotransferase (AST) and alanine aminotransferase (ALT) levels.

#### 2.3.3 Nitrogen balance assessment

For a subset of patients where feasible, 24-hour urine collections were performed to measure urinary urea nitrogen (UUN). Nitrogen balance was calculated using the standard formula: Nitrogen Balance (g/day) = [Protein Intake (g/day)/6.25] - [24-hour UUN (g/day) + 4], where 4 represents estimated non-urea nitrogen losses.

#### 2.3.4 Clinical symptom monitoring

Participants were monitored throughout the study period for the occurrence and severity of potential gastrointestinal symptoms related to the supplement, including nausea, vomiting, diarrhea, and constipation. Tolerance was assessed based on patient reporting and clinical observation.

#### 2.3.5 Dietary intake evaluation

A 24-hour dietary recall method was employed at baseline and endpoint to estimate total daily caloric and macronutrient intake from the regular hospital diet, allowing for comparison of overall intake before and during the supplementation period.

### 2.4 Intervention

Following the initial comprehensive assessment and determination of individual caloric needs, participants commenced supplementation with the Complete Nutrition Diabetic Formula. The prescribed daily dosage varied between 2 and 8 packs (each pack containing 60 g powder), tailored to meet the estimated individual energy and protein requirements not covered by voluntary oral intake from the standard hospital diet. Participants integrated the supplement into their daily routine according to personal preference and eating habits (e.g., as snacks between meals, meal replacements if oral intake was poor, or mixed into other foods/beverages). The average daily supplement intake across the cohort was 2.6 packs. Adherence was ensured through a dual-step process: ward nurses dispensed each participant’s prescribed sachets daily, logged the exact intake time, and retrieved empty packs, while a study dietitian cross-checked these records and reconciled any discrepancies via same-day patient interviews. Compliance was defined as consuming at least 80% of prescribed sachets, calculated from the ratio of packs consumed to packs prescribed. Missed doses were re-offered later the same day and, if still declined, the reason was documented (e.g., scheduled fasting or nausea). The duration of supplementation varied based on the length of hospital stay and clinical need, ranging from a minimum of 14 days to a maximum of 67 days, with an average intervention period of 29 days. Throughout the supplementation phase, participants continued to receive standard medical care for their diabetes and other underlying conditions, including adjustments to hypoglycemic medications as clinically indicated. At the end of the supplementation period (or upon hospital discharge if earlier, provided the minimum duration was met), the nutritional and biochemical evaluations were repeated.

### 2.5 Data analysis

All collected data were entered into a database and analyzed Sigmaplot statistical software (SPSS version 10 for windows; SPSS Inc, Chicago). Continuous variables were assessed for normality of distribution. “Normality of the differences between baseline and endpoint values was assessed using Shapiro-Wilk tests (α = 0.05). For outcomes where normality was satisfied, paired t-tests were used. For outcomes violating the normality assumption, Wilcoxon signed-rank tests were employed.” Descriptive statistics were calculated, and results are presented as Mean ± Standard Deviation (SD). To evaluate the impact of the nutritional supplementation, paired t-tests were employed to compare pre-intervention (baseline) and post-intervention (endpoint) values for all measured parameters, including anthropometric indices (MAC, MAMC, TSF, BMI, etc.), biochemical markers (fasting glucose, serum magnesium, albumin, lipids, etc.), and calculated nitrogen balance. A p-value of less than 0.05 was considered statistically significant for all comparisons.

## 3 Results and discussion

### 3.1 Baseline characteristics and supplementation

**Figure 2:**
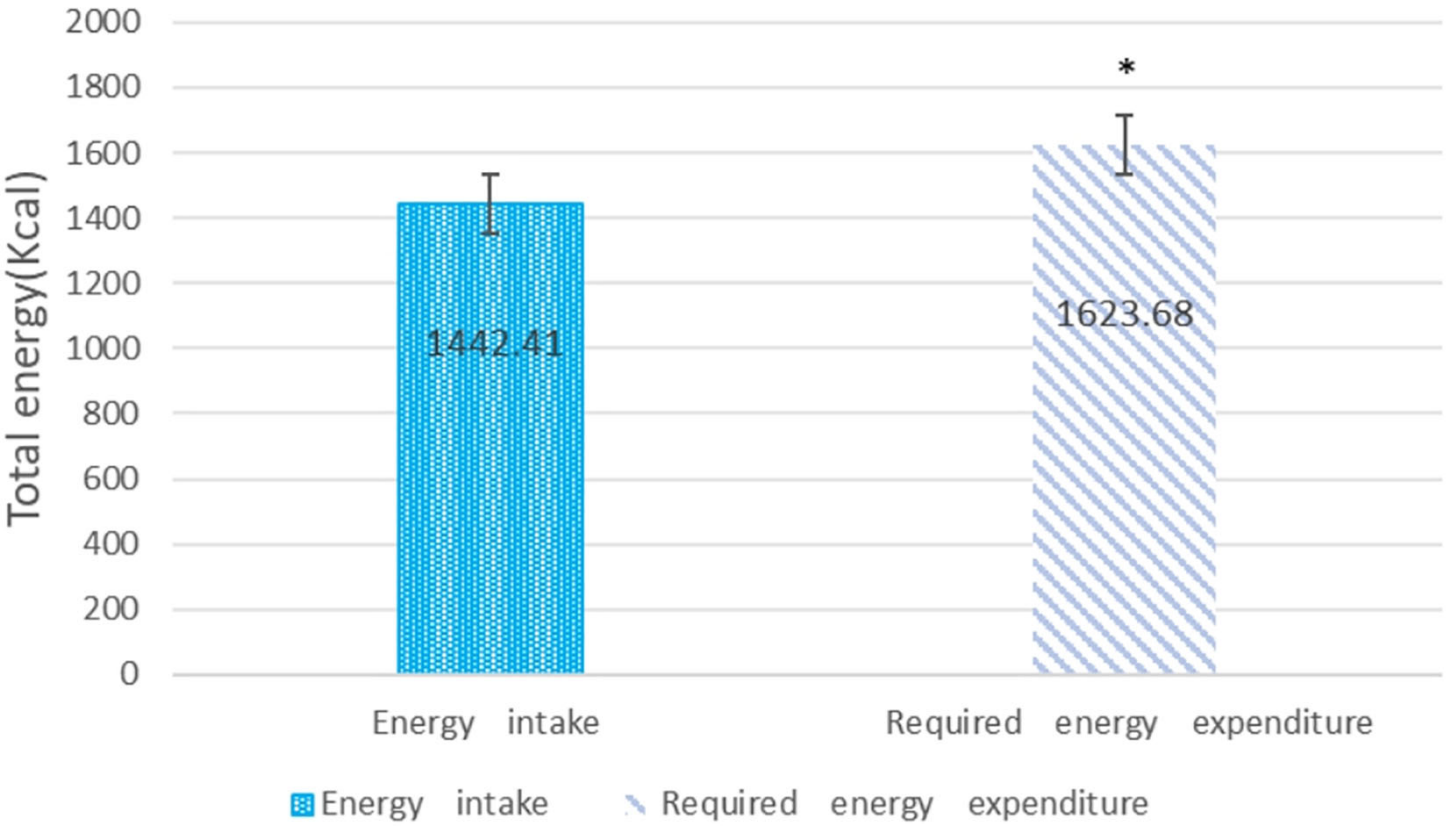
Assessment of caloric intake before supplementation with complete nutrition diabetic formula compared to estimated energy requirements. Data are means ± SD (n=40) *p<0.05, between energy intake and energy expenditure.

The study included 40 hospitalized diabetic patients (mean age 63 years). Supplementation with the Complete Nutrition Diabetic Formula was provided for an average of 29 days. Prior to supplementation with the Complete Nutrition Diabetic Formula, participants’ caloric intake was significantly lower than their estimated energy requirements (*p<0.05*) (**Figure 2**). This finding, indicating that participants had significantly inadequate caloric intake compared to their estimated needs before starting the nutritional supplement, is highly consistent with previous research on hospitalized patients, particularly those with diabetes mellitus.

### 3.2 Impact on anthropometric measures and nutritional status

A key finding (**Table 1**) was the significant increase in mid-arm circumference (MAC) post-supplementation (p<0.05). This aligns with the established role of MAC as a practical indicator of peripheral tissue reserves and its validity in assessing nutritional status and predicting outcomes in hospitalized patients, including those where weight/BMI may be unreliable due to fluid shifts (5). The observed increase suggests an improvement in somatic protein and energy stores, likely reflecting the positive nitrogen balance achieved. However, research suggesting that protein supplementation can positively impact skeletal muscle mass through enhanced muscle protein synthesis provides indirect support for potential MAC increases in diabetic individuals (15).

**Table 1:** Anthropometric measurements before and after supplementation with complete nutrition diabetic formula.

Variable	Before	After
Body height (cm)	160.37 ± 7.99^a^	160.37 ± 7.99^a^
Body weight (Kg)	61.65 ± 8.40^a^	61.86 ± 8.00^a^
BMI (Kg/m²)	24.03 ± 3.49^a^	24.12 ± 3.34^a^
SBP (mm Hg)	126.05 ± 12.15^a^	128.05 ± 10.72^a^
DBP (mm Hg)	78.85 ± 8.68^a^	79.20 ± 8.22^a^
WC (cm)	84.72 ± 12.15^a^	84.78 ± 12.09^a^
RC (cm)	93.64 ± 12.09^a^	93.60 ± 12.00^a^
WC/RC	0.90 ± 0.08^a^	0.91 ± 0.08^a^
TSF (mm)	17.51 ± 8.75^a^	17.49 ± 8.72^a^
MAC (cm)	27.77 ± 2.85^a^	27.85 ± 2.87^b^
MAMC (cm)	21.87 ± 3.95^a^	21.92 ± 3.94^a^

Data are means ± SD (n=40) *Different letters indicate significant differences (p<0.05) between before and after supplementation.

BMI, body weight (kg)/body height (m²); SBP, systolic blood pressure; DBP, Diastolic blood pressure; WC, waist circumference; RC, rump circumference; TSF, triceps skinfold; MAC, Midarm circumference; MAMC, Midarm muscle circumference.

An upward trend in body weight and Body Mass Index (BMI) was noted, although it did not reach statistical significance. This contrasts with findings from a meta-analysis on liraglutide (a pharmacological agent) which showed significant weight loss in diabetic patients, highlighting potential differences in response based on intervention type and population characteristics (16). Furthermore, studies on probiotic/synbiotic supplementation reported only minor weight reduction (0.5 kg) and limited BMI decrease (-0.24 kg/m^2^), differing from the observed upward trend (17). Personalized nutrition therapy combined with glucose monitoring has been linked to weight loss and improved body composition, but the specific direction of BMI change was not always detailed (18).

No significant changes were found in waist circumference (WC), hip circumference (HC), or waist-to-hip ratio (WHR). This observation aligns with findings from an 8-week krill oil supplementation trial in overweight women, which also reported no significant alterations in WC or sagittal abdominal diameter (19). Conversely, some dietary interventions have shown significant WC reductions, although factors like smaller sample sizes might contribute to discrepancies (20). Meta-analyses indicating average WC reduction can be complicated by high heterogeneity among studies, potentially obscuring no findings in specific subgroups or interventions (17).

Triceps skinfold thickness remained unchanged following the intervention. Supporting literature focusing specifically on skinfold changes in diabetic adults post-supplementation is sparse; one study evaluated nutritional supplements in preadolescent boys without providing relevant data for this population (21), and another discussed ultrasound for muscle assessment but did not report on skinfold measurements (22).

In summary, the effects of nutritional interventions on anthropometry appear variable, influenced by supplement type, duration, and patient characteristics. Localized improvements, such as MAC, might manifest earlier than systemic changes like weight or BMI, especially in shorter interventions. The absence of significant changes in central adiposity measures could relate to fat distribution patterns or measurement sensitivity. It is noteworthy that direct literature evidence perfectly matching the combination of observed changes (e.g., increased MAC with non-significant weight gain) was limited in the reviewed studies.

### 3.3 Impact on biochemical and hematological parameters

For **Table 2**, no significant alterations were observed in serum albumin or prealbumin levels (p>0.05). While preclinical models show that certain interventions, like the drug celastrol, can improve reduced albumin levels in diabetic nephropathy models, the relatively short duration (14-67 days) of the nutritional supplementation in the current observation might be insufficient to induce significant changes in albumin, especially if the supplement lacked specific protein or anti-inflammatory components.

**Table 2:** Changes in blood biochemical parameters before and after supplementation with complete nutrition diabetic formula.

Variable	Before	After
Albumin (g/dL)	4.18 ± 0.64^a^	4.17 ± 0.63^a^
Prealbumin (mg/dL)	29.52 ± 7.39^a^	28.54 ± 8.03^a^
TG (mg/dL)	187.58 ± 110.68^a^	169.63 ± 71.20^a^
Cholesterol (mg/dL)	200.43 ± 52.90^a^	197.83 ± 42.48^a^
BUN (mg/dL)	16.82 ± 7.67^a^	17.97 ± 8.45^a^
Glucose (mg/dL)	132.10 ± 53.55^a^	139.05 ± 68.13^a^
GOT (U/L)	22.66 ± 9.96^a^	25.13 ± 12.50^a^
GPT (U/L)	24.89 ± 18.19^a^	26.34 ± 20.02^a^
Creatinine (mg/dL)	1.45 ± 2.69^a^	1.66 ± 3.87^a^
Uric acid (mg/dL)	5.93 ± 1.72^a^	5.71 ± 1.93^a^
Hb (g/dL)	13.93 ± 2.57^a^	13.90 ± 1.52^a^
RBC (10^6^/uL)	4.48 ± 0.90^a^	4.43 ± 0.78^a^
WBC (10³/uL)	6.63 ± 1.71^a^	6.86 ± 1.73^a^
Na (mmol/L)	139.68 ± 3.60^a^	138.96 ± 2.70^a^
K (mmol/L)	4.58 ± 1.22^a^	4.29 ± 0.54^a^
Fe (ug/dL)	85.88 ± 35.29^a^	86.05 ± 26.97^a^
Ca (mg/dL)	9.87 ± 1.84^a^	9.39 ± 0.32^a^
Mg (mg/dL)	2.25 ± 0.20^a^	2.31 ± 0.20^b^
P (mg/dL)	3.74 ± 0.65^a^	3.89 ± 1.9^a^

Lipid metabolism markers, specifically Triglycerides (TG) and Total Cholesterol (TC), showed no significant changes. This finding contrasts with multiple studies demonstrating that specific interventions, such as probiotic supplementation or the use of plant extracts like celastrol or α-ketoacid analogues (KAA), can significantly lower TG and TC levels in diabetic populations. The lack of effect observed here might stem from the specific type of supplement used (i.e., one not containing these targeted lipid-lowering agents) or potentially an insufficient duration of the intervention to manifest lipid profile changes (23).

Blood glucose and liver enzyme levels remained stable. Glycemic control often necessitates longer-term or more targeted interventions, although minerals like zinc and magnesium have shown potential over time to improve insulin sensitivity (24, 25). Similarly, while liver enzymes (ALT, AST) can be elevated in diabetes and potentially improved by antioxidant supplements (e.g., Vitamin E, selenium), the observed stability might indicate the supplement did not specifically target liver protection or that baseline levels were not elevated (26). The lack of glucose change could also reflect a supplement formulation not directly targeting glucose metabolism pathways, perhaps lacking sufficient chromium or zinc (27).

Hemoglobin and red blood cell counts did not change significantly. Anemia is a known comorbidity in diabetes, but improvements typically necessitate targeted nutritional support, including iron, vitamin B12, or folate, or pharmacological interventions stimulating erythropoiesis. The stable hematological parameters observed likely suggest that the nutritional supplement provided did not contain these specific hematinic nutrients in sufficient amounts to effect change (28).

#### 3.3.1 Serum magnesium

Serum magnesium levels significantly increased (p<0.05), while other measured minerals remained unchanged (p>0.05). The rise in magnesium aligns well with literature indicating that diabetic patients often exhibit lower magnesium levels and that supplementation can effectively correct this deficit, potentially improving insulin sensitivity and even HDL-C levels (though HDL was not reported here) (25). The lack of change in other minerals like zinc or calcium is unsurprising, as significant alterations in their serum levels usually require targeted, specific supplementation, sometimes with necessary co-factors like Vitamin D for calcium absorption (29–32).

Renal function markers showed a decrease in Blood Urea Nitrogen (BUN) alongside stable creatinine levels (p<0.05) (**Table 3**). This pattern suggests preserved or potentially improved renal function, consistent with findings that interventions like probiotics, KAA, or certain plant extracts can lower BUN (32). This contrasts with some diabetic nephropathy models where BUN and creatinine often rise concurrently (33), suggesting the observed BUN decrease might reflect improved nitrogen metabolism possibly mediated by the specific nutritional intervention provided.

**Table 3:** Changes in urinary biochemical parameters before and after supplementation with complete nutrition diabetic formula.

Variable	Before	After
UUN (mg/dL)	434.12 ± 147.02^a^	453.62 ± 157.65^a^
Urine Ca (mg/L)	15.8 ± 3.45^a^	15.12 ± 3.22^a^
Volume (mL)	2199.16 ± 787.99^a^	2248.88 ± 846.22^a^
Total Urine Ca (mg)	167.14 ± 114.42^a^	170.28 ± 117.46^a^
24 hr UUN (g)	7.77 ± 2.74^a^	7.98 ± 3.26^a^

Data are means ± SD (n=40).

*Different letters indicate significant differences (p<0.05) between before and after supplementation.

In summary, the biochemical and hematological findings present a mixed picture. The significant increase in magnesium and decrease in BUN align with potential benefits reported in the literature for specific interventions. However, the lack of change in albumin, lipids, glucose, liver enzymes, and other minerals likely reflects the specific composition and duration of the nutritional protocol, possibly lacking targeted components for these parameters. Future studies might benefit from longer durations and the inclusion of specific nutrients like zinc, chromium, probiotics, or antioxidants to achieve broader metabolic improvements.

### 3.4 Impact on dietary intake and nitrogen balance and glycemic control

Post-supplementation dietary analysis confirmed significantly increased total caloric and fat intake, with a reduced carbohydrate percentage, reflecting the DSF’s composition (**Table 4**). Crucially, nitrogen balance shifted significantly from negative (-2.7 g/day) to positive (+1.25 g/day) (p<0.05). Vitamin D supplementation, particularly when used with anti-diabetic drugs, has been shown to enhance glycemic control and potentially reduce complication risks in T2DM, supporting its role as an adjunct therapy, though studies typically do not report on nitrogen balance outcomes (34).

**Table 4:** Changes in calorie and macronutrient intake before and after supplementation with complete nutrition diabetic formula.

Variable	Before	After
Total energy (Kcal)	1442.41 ± 347.49	1720.54 ± 336.56*
Carbohydrate (g)	178.69 ± 46.20	160.28 ± 36.12*
Protein (g)	56.68 ± 15.93	81.44 ± 12.35*
Lipid (g)	54.09 ± 19.07	83.74 ± 17.58*

Data are means ± SD(n=40) *p<0.05,between before and after supplementation.

Systematic reviews and network meta-analyses comparing various micronutrients like Vitamins D, C, E, and magnesium have focused primarily on their effects on glycemic and lipid control in T2DM. While these analyses suggest modulatory roles for micronutrients, they lack specific data on nitrogen balance and conclude that more evidence is needed to establish definitive comparative efficacy for glycemic management (35). Research exploring probiotic supplementation in the context of diabetic nephropathy highlights potential benefits for renal function, inflammation reduction, and possibly glycemic control. However, these studies generally do not include measurements or analyses related to nitrogen balance changes (36).

Preclinical studies using zinc supplementation in diabetic rat models (ZDF rats) demonstrated improvements in obesity, glycemic control, pancreatic function, and reductions in liver steatosis and kidney damage. While not directly measured, these broad metabolic improvements suggest zinc could indirectly influence nitrogen balance via enhanced overall metabolic regulation and tissue health (37). Studies focusing on amino acids in diabetes emphasize the importance of monitoring levels and have explored the impact of specific amino acids like serine and glycine on disease progression. Some research suggests amino acid supplementation might associate with lower mortality, but direct reporting on nitrogen balance changes or its link to glycemic control in these interventions is typically absent (38, 39).

The use of specialized enteral nutrition (EN) formulas designed for diabetes has been shown to provide superior glycemic control compared to standard formulas, often leading to reduced insulin needs. Case series and trials report improved clinical indicators and compare glycemic/insulinemic responses, but specific analysis of nitrogen balance changes resulting from these diabetes-specific EN formulas is often not a primary endpoint (40, 41). Similarly, investigations into diabetes-specific oral nutritional supplements (ONS), such as those containing alternative sweeteners like D-allulose, have demonstrated favorable anti-hyperglycemic effects and good safety profiles in overweight or obese T2DM patients. However, these studies typically focus on glycemic and weight outcomes and do not include nitrogen balance assessments (42).

Non-significant findings may reflect Type II error due to insufficient sample size rather than true metabolic neutrality. Conclusions regarding safety should be tempered by the study’s limited power to detect small-moderate effects.

The observed significant improvement in nitrogen balance (from negative to positive) is metabolically plausible with improved nutritional intake but lacks direct corroboration within the specific studies cited. While some interventions involving zinc or amino acids hint at potential mechanisms for improved nitrogen retention through better metabolic control, direct evidence is scarce. Conversely, the observation of stable or potentially improved glycemic control aligns well with the literature, which generally supports the safety or benefit of various nutritional supplements (like Vitamin D, probiotics, specialized formulas) in managing blood glucose levels. A notable research gap exists regarding studies that concurrently measure macronutrient intake changes, nitrogen balance, and glycemic responses following specific nutritional supplementations in diabetic patients. Further targeted research is needed to validate the impact of nutritional strategies on nitrogen balance and understand its interplay with glycemic control in this population.

This study provides valuable clinical data on the integrated effects of a specific Complete Nutrition Diabetic Formula in hospitalized diabetic patients, addressing a gap identified in the introduction. The findings demonstrate that supplementation significantly improved key nutritional markers – MAC, serum magnesium, and nitrogen balance – indicating enhanced somatic stores, correction of a common micronutrient deficit, and a shift towards anabolism. These improvements occurred concurrently with the expected dietary shift induced by the DSF. Critically, and consistent with extensive prior research on DSFs, these nutritional benefits were achieved without compromising fasting glycemic control, highlighting the metabolic advantage of this specialized formula over standard options ^5^ in this high-risk population. The formula was well-tolerated with no adverse effects on measured renal, hepatic, or lipid parameters.

The results support the use of this DSF as a safe and effective tool for managing the dual challenge of malnutrition and glycemic control in hospitalized diabetic patients. The improvement in MAC ^10^ and nitrogen balance signifies effective nutritional repletion, while the increase in serum magnesium addresses a specific deficiency prevalent in diabetes.

Limitations include the single-center, open-label design, the variable supplementation duration, and reliance on fasting glucose rather than more comprehensive glycemic monitoring. Future studies of longer duration or with a primary glycaemic focus should incorporate HbA1c and structured post-prandial monitoring to characterise fully the metabolic impact of DSFs. Lack of a control group receiving a standard formula limits direct comparison within this study, although the comparison to baseline and established literature on SF vs DSF provides context. Future larger, controlled trials with longer follow-up are warranted to confirm these findings and assess impacts on clinical outcomes like length of stay or readmissions, which are known to be affected by malnutrition. In addition, unadjusted p-values are reported for exploratory outcomes; significant results require validation in adequately powered trials. Non-significance in some parameters (e.g., weight, lipids) may reflect limited statistical power.

## 4 Conclusion

Supplementation with the ‘Complete Nutrition Diabetic Formula’ in hospitalized diabetic patients effectively improved key indicators of nutritional status, including mid-arm circumference, serum magnesium levels, and nitrogen balance, over an average of 29 days. Consistent with the properties of diabetes-specific formulas documented in previous research, these nutritional benefits were achieved without compromising fasting blood glucose control. Observed stability in glucose, lipids, and renal markers over the intervention period suggests no significant short-term detriment within this cohort, though larger controlled studies are needed to confirm safety. These findings support the clinical utility of this specific DSF as part of medical nutrition therapy for managing the complex interplay of nutritional needs and glycemic stability in this vulnerable inpatient population.

## Data Availability

The original contributions presented in the study are included in the article/supplementary material. Further inquiries can be directed to the corresponding authors.
